# Exosomal Lipids in Cancer Progression and Metastasis

**DOI:** 10.1002/cam4.70687

**Published:** 2025-03-20

**Authors:** Dandugudumula Ramu, Eunjoo Kim

**Affiliations:** ^1^ Division of ABB Research Daegu Gyeongbuk Institute of Science and Technology (DGIST) Daegu Republic of Korea

**Keywords:** cancer metastasis, exosomes, extracellular vesicles, lipids, oncogenic process

## Abstract

**Background:**

Metastasis is the primary cause of cancer mortality. It is responsible for 90% of all cancer‐related deaths. Intercellular communication is a crucial feature underlying cancer metastasis and progression. Cancerous tumors secrete membrane‐derived small extracellular vesicles (30–150 nm) into their extracellular milieu. These tiny organelles, known as exosomes, facilitate intercellular communication by transferring bioactive molecules. These exosomes harbor different cargos, such as proteins, nucleic acids, and lipids, that mediate multifaceted functions in various oncogenic processes. Of note, the amount of lipids in exosomes is multifold higher than that of other cargos. Most studies have investigated the role of exosomes' protein and nucleic acid content in various oncogenic processes, while the role of lipid cargo in cancer pathophysiology remains largely obscure.

**Materials and Methods:**

We conducted an extensive literature review on the role of exosomes and lipids in cancer progression, specifically addressing the topic of exosomal lipids and their involvement in cancer metastasis and progression.

**Conclusions:**

This review aims to shed light on the lipid contents of exosomes in cancer metastasis. In this context, the role of exosomal lipids in signaling pathways, immunomodulation, and energy production for cancer cell survival provides insights into overcoming cancer progression and metastasis.

## Graphical abstract

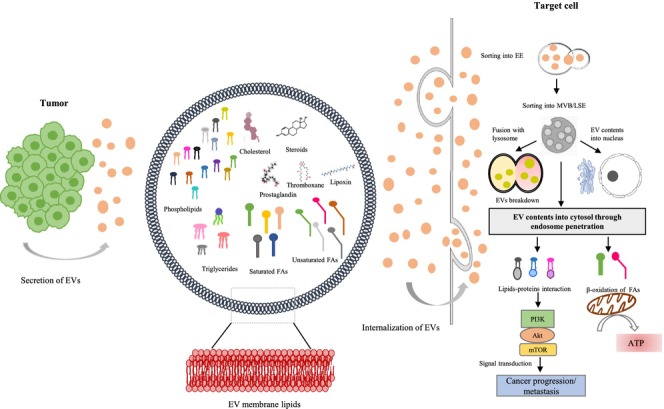

## 1 Introduction

Cancer is one of the leading causes of human deaths worldwide and is a formidable challenge in global health [1]. Cancer metastasis is one of the most fearsome aspects of cancer. Essentially, metastasis is described as the migration of tumor cells from the primary tumor, followed by the progressive colonization of distant organs. Since the precise mechanism behind cancer metastasis remains largely obscure, understanding this underlying molecular mechanism is crucial for containing metastatic dissemination and the progression of cancer.

Lipids, the primary class of biomolecules that serve as critical structural components of cells, also play a crucial role in signaling pathways. Lipids contribute to the structural integrity of cells and are involved in cell signaling and energy. Lipids have been found to contribute to cancer progression and metastasis, and metabolic reprogramming of lipids has emerged as a critical hallmark of cancer [2, 3]. Highly proliferative cancer cells enhance lipid avidity by upregulating lipid synthesis through the de novo pathway or increasing lipids or lipoproteins uptake [4]. These bioactive lipids act as secondary signaling messengers, coordinating signal transduction cascades and various carcinogenic processes, such as cell survival, proliferation, angiogenesis, and immunomodulation. Moreover, lipid autacoids such as prostaglandins (PGs), prostacyclin, thromboxane A2, and leukotrienes are involved in intercellular communication between noncancerous and cancerous cells. These lipid autacoids comprise signaling lipids and target different cellular components in tumor microenvironments [5]. During metastasis, tumor cells undergo various stages that require lipids to mediate structural and metabolic adaptations [6]. Therefore, targeting lipids involved in cancer metastasis provides a promising approach to developing robust cancer therapeutic interventions due to their critical role in cancer progression.

Exosomes have emerged as novel mediators in cell–cell communication and have gained significant attention in recent times [7]. Several types of tumors release exosomes, transport a variety of cargo out of parent cells, and deliver them to nearby or distant cells/tissues, correlating with tumor microenvironment and tumor dissemination, such as pre‐metastatic niche formation, angiogenesis promotion, immunomodulation, and drug resistance. One of the most intriguing aspects of exosomes is the wide variety of cargo they can carry, including proteins, nucleic acids, and lipids, which mediate multifaceted functions in cancer pathogenesis [8]. Of note, exosomes' lipid content is multiple folds higher than that of protein and nucleic acids cargo. For instance, exosomes derived from prostate cancer cells (PC3) revealed an overall 8.4‐fold enrichment of lipids per mg of protein in cancer cell‐derived exosomes [9]. However, the role of lipid cargo in cancer pathogenesis largely remains unknown [10, 11]. Along the same line, being a principal constituent of exosomal cargo, exploring the lipid content of exosomes may help uncover the mechanisms behind cancer metastasis. This review aims to highlight the significance of lipids in different oncogenic processes, with particular emphasis on the lipid cargo of exosomes in cancer progression and metastasis.

## 2 Significance of Lipids in Cancer Progression and Metastasis

Lipids are a diverse class of biomolecules consisting of fatty acids (FAs) and their derivatives, that are readily soluble in organic solvents but insoluble in water. Lipids are structural components constituting cell membranes and act as signaling molecules that regulate various cellular functions. Lipids also serve as fuel to drive energy‐demanding processes [12, 13, 14].

### 2.1 Lipid Metabolism in Cancer

Abnormal lipid metabolism is the primary metabolic alteration observed in cancer cells, which can have both pro‐tumorigenic and anti‐tumorigenic effects [15]. In cancer cells, lipid metabolism is regulated by extracellular lipids and endogenous lipogenesis. For example, Marino et al. observed the simultaneous adipogenesis and upregulation of genes involved in lipid metabolism in breast cancer tumors. Increased phospholipids (PLs) are involved in bioactive lipid synthesis, a second messenger that modulates membrane lipid composition. Consequently, they play a role in metastasis and drug resistance [16, 17]. For instance, specific precursors of PLs and cholesterol, including ceramides and isoprenoids, trigger multiple signaling cascades by acting as second messengers, which facilitate metabolic reprogramming and/or induce the transcription of drug efflux transporter genes, leading to the multidrug resistance phenotype in tumor cells.

Increased lipid synthesis and uptake result in an increase of the cellular lipid pool of tumors. These lipids are stored within lipid droplets (LDs), which are derived from the monolayer membrane of the endoplasmic reticulum (ER) and encapsulate neutral lipids internally. LDs can extensively mediate tumor progression, metastasis, and chemotherapy resistance in various cancers. LDs play a role in maintaining ER homeostasis by reducing the aggregation of unfolded and damaged lipids, thus inhibiting ER stress and promoting cancer cell proliferation [18, 19, 20, 21, 22, 23, 24]. High metabolism in cancer cells leads to the production of reactive oxygen species (ROS). Moreover, during hypoxia, cancer cells upregulate FAs uptake, and LDs protect cells from ROS by supporting NADPH synthesis [25]. This clearance of ROS accumulation by LDs promotes drug resistance in cancer cells. LDs serve as a critical reservoir of unsaturated FAs, enabling a homogeneous distribution of FAs into tubulated mitochondria and maximizing the efficiency of FA oxidation during starvation, which is a critical energy source for cancer cells under nutrient‐deprived conditions. Furthermore, the crosstalk between LDs and intracellular free FAs facilitates tumor growth and sustains immunity and inflammation in the tumor microenvironment [26, 27]. Overall, these roles of LDs in oncogenesis suggest that the inhibition of LD anabolism and catabolism plays a crucial role in cancer therapeutics.

Fatty acid oxidation (FAO) is an energy production pathway for tumor cells under nutrient‐deficient conditions in cancer progression. This contradicts the well‐known Warburg effect, which suggests aerobic glycolysis as the primary energy source for tumor cells [28, 29]. FAO is the catabolic process for breaking down FAs to generate acetyl‐CoA, NADH, FADH2, and ATP in the mitochondria. Recent reports indicate that ATP production in cancer cells mainly depends on the FAO of mitochondria [30]. Table 1 summarizes the changes in lipid‐related metabolism in cancer progression, such as increased lipid synthesis, uptake, storage, and FAO.

**TABLE 1:** The role of lipids and lipid‐derived molecules in various oncogenic processes.

Lipids or lipid‐derived molecules	Role of cancer	References
Sphingolipids	Promotes proangiogenic factors in the TME	61
Leukotrienes	Establishes a pre‐metastatic environment	92,93
Fatty acids	Serves as an energy source for tumor cells though β‐oxidation	111,1112,113
Phospholipid (PL)	Bioactive lipids synthesis, modulation of membrane lipid composition for metastasis and drug resistance	57,58,75
Lysophosphatidic acid (LPA)	Signaling molecules for TME	59, 60
Prostaglandin (PG)	Signaling molecules for TME	123, 124
Lipid droplets (LD)	Cancer cells protection during oxidative stress	59, 60
Phosphatidic acid (PA)	Involved in pro‐inflammation	66
Ceramide	Involved in bone marrow‐derived macrophages (BMDMs) apoptosis in TME	67
Docosahexaenoic acid (DHA)	A ω‐3 fatty acid involved in inflammation	68
Plasmalogens	Regulation of cancer cells survival, growth and aggressiveness	69
Lipid‐associated lncRNAs	Cancer signal transduction	70, 71. 72

### 2.2 Lipids‐Mediated Cell Signaling in Cancer

In addition to their metabolic and structural roles, lipids function as signaling molecules within and between cells. For example, membrane PLs are converted into lipid mediators like phosphatidic acid (PA), diacylglycerol (DAG), arachidonic acid, and lysophosphatidic acid (LPA) through the activity of phospholipases(PLs) [31]. Some of these, such as arachidonic acid, are further transformed into leukotrienes and PGs via lipoxygenase and cyclooxygenase pathways, respectively. These active lipids can be released from cancer cells and act as autocrine or paracrine messengers, involving different oncogenic processes that promote tumor progression and metastasis.

For instance, LPA and PGs enhance the release of angiogenic cytokines and vascular endothelial growth factor (VEGF) to facilitate angiogenesis. Furthermore, these active lipid mediators, particularly PGs, can also drive chronic inflammation, which promotes tumor growth and can impact stromal cells. Conversely, cancer‐associated fibroblasts (CAFs) release lysophosphatidylcholines (LPCs) [32], which cancer cells convert into LPA, thereby promoting proliferation and migration through the activation of AKT signaling. Additionally, membrane sphingolipids, such as ceramides and sphingosine‐1‐phosphate, function as bioactive molecules with significant roles in cancer regulation, exhibiting primarily anti‐proliferative and pro‐survival signaling effects, respectively.

Palmitoylation is one of the major post‐translational modifications (PTMs) of proteins involved in oncogenic processes. This lipidation of proteins plays a crucial role in various oncogenic signaling pathways, such as Ras, epidermal growth factor (EGF), Wnt, and Hippo pathways [33]. For example, the Ras family of GTPases can undergo S‐palmitoylation. Blocking the S‐palmitoylation of the oncogenic NRASG12D mutant at Cys181 causes mislocalization of the mutant NRAS, inhibiting downstream signaling, which hinders disease development and extends the lifespan of leukemia‐bearing animals [34]. Similarly, S‐palmitoylation of EGFR can promote ligand‐independent homodimerization and activation, a process that appears to rely on FASN activity. This modification can relocate EGFR to the nucleus, contributing to chemotherapy resistance [35, 36].

Moreover, cholesterol plays a crucial role in the Hedgehog (Hh) signaling pathway, which plays an instructional role in organogenesis and tissue homeostasis [37, 38]. Before secretion, HH protein undergoes PTMs that involve cholesterol addition. Cholesterol is required to activate Smoothened, a G‐protein coupled receptor (GPCR) that transmits the Hedgehog signal across the cell membrane. Accessible cholesterol in the cell membrane acts as a second messenger, facilitating the interaction between Smoothened and Patched1 (Ptch1), another receptor involved in the pathway, which is crucial for HH signal transduction [39, 40, 41]. Thus, cholesterol is essential for HH signal transduction and regulation, and aberrant Hedgehog signaling has been reported in various types of cancer.

Plasmalogens are a subclass of glycerophospholipids characterized by the presence of a vinyl‐ether bond at the sn‐1 position of the glycerol backbone. Plasmalogens are linearly correlated with several oncogenic signaling lipids that regulate tumor aggressiveness and growth [42]. Plasmalogens have been found to activate phosphatidylinositol 3‐kinase, promote cell growth, and participate in mitogenic responses. They are also linked to oncogenic signaling lipids that regulate cell survival, cancer aggressiveness, and tumor growth [43]. Additionally, it was suggested that a potential link exists between plasmalogens and colorectal cancer (CRC), hypothesizing that the membrane fluidity provided by plasmalogens may aid in capturing carcinogenic substances, both microbial and dietary. Chromatographic analysis of colorectal carcinoma phospholipid content revealed generally elevated plasmalogen levels, including a specific PE plasmalogen species (34:2) [44, 45].

Long non‐coding RNAs (lncRNAs) predominantly reside in the nucleus and are involved in diverse biological processes. However, recent studies have proposed emerging roles for cytoplasmic lncRNAs, including those associated with membrane lipids. These lncRNAs influence cancer metastasis and progression by modulating cancer signaling pathways via lipid‐related cellular functions. So far, several lipid‐associated lncRNAs, including small nucleolar RNA host gene 3 (SNHG3), small nucleolar RNA host gene 6 (SNHG6), and LINK‐A (formerly LINC01139), have been found to be involved in tumor progression [46, 47]. Lin et al. demonstrated that SNHG6 contributes to tumor growth and metastasis of hepatocellular carcinoma (HCC) through epithelial‐to‐mesenchymal transition [48]. This tumorigenesis is caused by the binding of SNHG6 to up‐frameshift protein 1 and the regulation of Smad7 expression. Additionally, Cao et al. found that SNHG6‐003 modulates the expression of transforming growth factor‐β‐activated kinase 1 (TAK1), and the upregulation of SNHG6‐003 and TAK1 correlates with tumor progression in human cancers. Lin et al. also identified a cytoplasmic lncRNA, LINK‐A (long intergenic non‐coding RNA for kinase activation), which promotes the recruitment of BRK to the EGFR:GPNMB, BRK kinase. The BRK‐dependent phosphorylation of HIF1α at Tyr 565 interferes with Pro 564 hydroxylation, stabilizing HIF1α under normoxic conditions. Both LINK‐A expression and signaling pathway activation are correlated with triple‐negative breast cancer (TNBC) [49, 50]. Furthermore, the same group found that LINK‐A interacts with AKT and PIP3, causing AKT recruitment and activation, which leads to tumorigenesis and resistance to AKT inhibitors.

Overall, these observations strongly suggest that lipids are implicated in various cell signaling processes involved in oncogenic mechanisms to facilitate cancer progression and metastasis.

### 2.3 Modulation of Cancer Immunity by Lipids

Certain studies suggest that lipids such as sphingomyelin (SM), including ceramides, PAs, and docosahexaenoic acid (DHA), have immunomodulatory properties. SM plays a crucial role in cell processes, inflammatory responses, and signal transduction, and plays a broad role in oncogenic processes [51]. Several reports suggest that SM regulates cancer cell proliferation and invasion. In these studies, tumor progression has been correlated with the disturbed metabolic balance of SM and ceramide [52, 53]. Tumor‐associated macrophages participate in the regulation of interactions between cancer cells and the immune system [54, 55]. In this context, PA upregulates the expression of pro‐inflammatory molecules in macrophages by amplifying lipopolysaccharides (LPS) signaling [56]. Ceramide is also involved in bone marrow‐derived macrophage apoptosis, and it was found that sphingomyelinase activation and ceramide generation in bone marrow‐derived macrophages (BMDMs) induce apoptosis by growth factor withdrawal [57, 58]. DHA is a 3 fatty acid involved in inflammation. Impairment of systemic DHA synthesis delineates an alteration of M1/M2 macrophages, supporting the view that DHA plays a key role in controlling the balance between pro‐ and anti‐inflammatory processes [59]. The lipid‐rich tumor microenvironment also results in defective antitumor properties in dendritic cells (DCs). DCs, a type of professional antigen‐presenting cell, present tumor‐associated antigens that can cause a T cell‐dependent anticancer immunity. It was observed that DCs with high LD content were unable to present tumor‐associated antigens to stimulate a T cell response [32]. Given the diverse roles of lipids in cancer progression, exosome‐derived lipids could be recognized as an emerging factor contributing to the progression of cancer to metastatic states.

## 3 Exosomes Composition, Biogenesis, Release and Homing

### 3.1 Composition of Exosomes

Extracellular vesicles (EVs) are diverse, lipid‐bound vesicles secreted by all kinds of cells into various biological fluids and the extracellular space. EVs can be classified into different subpopulations based on their specific characteristics, such as biogenesis, size, composition, and functions, including apoptotic bodies (ABs), microvesicles (MVs), exosomes, and oncosomes [60, 61]. Exosomes are small extracellular vesicles, typically ranging from 30 to 150 nm in diameter, secreted by various cell types. Exosomal cargo mainly consists of lipids, small metabolites, proteins, and nucleic acids. All this cargo is selectively sorted into exosomes in a cell‐dependent manner.

As exosomes are distinct entities of complex cell–cell communication, their molecular composition depends on the parent cells and is often influenced by microenvironmental stimuli. Like cells, the surface of exosomes contains lipids such as cholesterol, phosphatidylserine (PS), ceramides, SM, and carbohydrate moieties. Besides lipids, membrane proteins such as Major histocompatibility complex (MHC) class II complexes, tetraspanins (TSPANs) (CD9, CD63, and CD81), ESCRT proteins, integrins, Rab GTPases (Rab4, Rab11, and Rab27), lactadherin (LA), and lysosome‐associated membrane protein‐2b (Lamp‐2b) are present on the surface of exosomes. These proteins can bind to specific ligands on recipient cells for stringent targeting ability.

Among extracellular vesicles, exosomes and MVs are regarded as primary vesicles. The different cargos of MVs vary from exosomes regarding their sorting and abundance. Concerning lipid cargo, Harastzi et al. performed high‐resolution lipidomic analyses of exosomes and MVs from U87 glioblastoma cells, Huh7 hepatocellular carcinoma cells, and human bone marrow‐derived mesenchymal stem cells (MSCs). It was found that exosomes and MVs also differed in their types of lipid content. Exosomes showed enrichment in glycolipids and free FAs, whereas MVs were characterized by enrichment in ceramides and SMs.

### 3.2 Biogenesis and Release Mechanisms of Exosomes

In the classical pathway, exosome biogenesis begins with the formation of the endosomal system, where early‐sorting endosomes (ESE) invade the inner endosome and bud to form multiple intraluminal vesicles (ILVs), consequently generating multivesicular bodies (MVBs). MVBs further develop into late‐sorting endosomes (LSEs) and fuse with the plasma membrane, eventually releasing internal ILVs into the external environment. Typically, MVB/ILV formation pathways are divided into two categories: the endosomal‐sorting complex required for transport (ESCRT) complex‐dependent pathway and the ESCRT‐independent pathway. The ESCRT complex consists of five different protein complexes, including ESCRT‐0, ‐I, ‐II, ‐III, and accessory proteins (AAA ATPase Vps4 complex), crucial for the formation of MVB/ILV [62, 63].

The role of lipids in the formation and secretion of exosomes in an ESCRT‐independent manner has been extensively studied. A set of studies showed that PL D2 (PLD2) controls the budding of MVBs and the development of exosomes, while DAG kinase (DGK) mediates the maturation and secretion of MVBs by regulating the subcellular localization and activation of PKD1/2 [64, 65]. It was found that the activity of neutral sphingomyelinase (nSMase), a ceramide‐producing enzyme, in exosomes derived from mouse oligodendrocytes is related to exosome biogenesis and release.

In the course of exosome release, MVBs undergo two “fates”: 1. MVBs fuse with lysosomes, causing the degradation of their contents. 2. MVBs fuse with the plasma membrane, leading to the release of ILVs and forming exosomes [66]. The transport of MVBs to the plasma membrane is essential for the release of exosomes and involves the microtubule cytoskeleton. The subsequent budding and release of MVBs depend on ATP‐driven contractions of cytoskeletal structures, specifically actin–myosin interactions. The release of ILVs from the lumen to the extracellular space requires MVBs to fuse with the plasma membrane, which is mainly performed by SNARE proteins, RABs, and Ras GTPase proteins [67, 68]. Further, calcium ions are also involved in this process, and a Ca^2+^ ‐dependent SNAP receptor and RAB‐binding protein promote the fusion of MVBs with the plasma membrane in an RAB11‐dependent manner [69, 70].

### 3.3 The Homing of Exosomes Is Critical for Targeting Ability

Exosomes exhibit efficient homing abilities, allowing them to carry a variety of cargo from their originating cells to nearby or distant cells/tissues [71]. For example, small exosomes derived from melanoma B16BL6 cells homed to the lungs within 10 min after their injection into animals [72]. This confirms the precision of exosomes in reaching their target destinations. The natural targeting ability of exosomes is based on the binding of exosomal membrane proteins to receptors on the surface of recipient cells.

The uptake of exosomes primarily depends on the recipient cells' signaling status and the composition of the exosomes. Membrane proteins such as MHC class II complexes, members of the TSPAN family (CD9, CD63, and CD81), ESCRT, integrins, Rab GTPases (Rab4, Rab11, and Rab27), lactadherin (LA), and lysosome‐associated membrane protein‐2b (Lamp‐2b) are abundantly present on the surface of exosomes. These proteins can interact selectively with target ligands to enhance their targeting ability [73, 74]. Additionally, lipids that mediate the binding of exosomes to recipient cells also play a role in the homing process.

Several investigations have suggested the involvement of TSPANs and integrins in the homing/tropism of exosomes. For instance, exosomes with high levels of TSPAN8 are preferentially taken up by the lungs and pancreas but rarely by the gut and liver. Interestingly, breast cancer stem cells exhibit increased expression of TSPAN8, which promotes the interaction of exosomes with their target cells through forced confined diffusion [75]. Furthermore, exosomes with gut‐homing ability show enriched integrins α4β7, highlighting the role of these molecules in exosomal tropism [76].

## 4 Exosomes Are the Key Modulators of Tumor Microenvironment

Bidirectional intracellular communication by exosomes contributes to cancer metastasis through the horizontal transfer of oncogenic molecules to the target cells [77, 78]. Epithelial‐to‐mesenchymal transition (EMT) is a crucial process for initiating metastasis. EMT involves the loss of epithelial cells' polarity and adhesion ability while gaining migratory and invasive properties, which generate mesenchymal stem cells [79, 80]. Exosomes derived from highly metastatic cells can induce the migratory ability of low‐metastatic cells by triggering the exosomes‐mediated EMT process via MAPK/ERK signaling [81]. Exosomes carry EMT‐related factors such as β‐catenin, caveolin‐1, TGFβ, and hypoxia‐inducible factor 1α(HIF‐1α) [82].

Hypoxia is regarded as a common feature of the tumor microenvironment, and hypoxia‐driven cancer progression is widely reported. Interestingly, exosomes released from tumor cells under hypoxic conditions are enriched with microRNA‐21 (miR‐21) and matrix metalloproteinase‐13(MMP‐13), causing metastases via EMT by increasing vimentin and decreasing E‐cadherin in normoxic cells. Shan et al. reported that exosomes from hypoxic nasopharyngeal carcinoma (NPC) cells enhance the metastasis of normoxic cells in a HIF‐1α‐dependent manner. Hypoxia‐inducible factor 1‐alpha (HIF‐1α) quickly builds up and activates numerous genes, including matrix metalloproteinases (MMPs). Under hypoxic conditions, MMP‐13 is highly expressed in both exosomes and cells, enhancing metastasis by inducing EMT in vitro and in vivo, increasing vimentin, and decreasing E‐cadherin in normoxic cells [82].

Primary tumors selectively modify the microenvironment of distant organs, a phenomenon termed pre‐metastatic niche formation [83, 84]. Cellular events like angiogenesis, immunosuppression, and metabolic reprogramming characterize the pre‐metastatic niche. Tumor‐derived exosomes are profoundly involved in forming pre‐metastatic niches [85, 86]. Angiogenesis, the creation of new blood vessels, plays a crucial role in tumor growth. Recently, the role of exosomes in angiogenesis has been a topic of interest in cancer research. Exosomes from breast cancer cells enriched with miR‐210 have been shown to induce angiogenesis by inhibiting EphrinA3 expression in vascular endothelial cells [87]. Exosomes derived from metastatic breast cancer tumors enriched with ANX II trigger angiogenesis. Another study showed that exosomes from squamous cell carcinoma promote angiogenesis by causing Ephrin‐B reverse signaling [88]. Overall, metastatic cancer‐derived exosomes are involved in angiogenesis, which is crucial for tumor development.

Metabolomic reprogramming is typically associated with transforming cancer cells into metastatic forms. Altered glucose metabolism is a hallmark of cancer [89]. It has been demonstrated that miR‐122 in exosomes secreted by breast cancer cells can transfer to normal cells in the pre‐metastatic niche, resulting in reduced glucose uptake by these cells [90]. Additionally, exosomes derived from cells infected with Kaposi's sarcoma‐associated herpesvirus can specifically transport viral miRNAs to neighboring cells, resulting in a metabolic switch towards aerobic glycolysis in recipient cells [91].

Numerous studies have shown the bidirectional exchange of lipids between various types of cancer cells and their surrounding adipocytes, which eventually promotes tumor progression [92] The cancer cell‐adipocyte interaction induces lipolysis in adipocytes and enables the transfer of FAs from adipocytes into cancer cells, leading to β‐oxidation in cancer cells. This phenomenon has been demonstrated in acute myeloid leukemia, ovarian, and prostate carcinoma [93, 94]. This interaction results in an increase in the production of lipases and fatty acid transport proteins in cancer cells, enabling them to use stored lipids for tumor growth. This lipid trafficking may be established by adipose tissue‐derived exosomes (AT‐Exosomes) mediated cell‐to‐cell communication between adipocytes and cancer cells within the microenvironment or systemically [95]. Adipose tissue serves as a reservoir of proinflammatory adipokines, such as tumor necrosis factor α (TNFα), interleukin (IL) 6, IL‐8, and chemokine (C‐C motif) ligand (CCL) 2, all of which are involved in tumor progression [96, 97]. Hartwig et al. demonstrated that adipokines are released either directly or via extracellular vesicles (Exosomes), referred to as exoadipokines. This group further observed that AT‐exosomes from obese individuals could cause a more aggressive phenotype of breast cancer cells through intracellular cell‐to‐cell communication [98]. Thus, AT‐exosomes facilitate various cell biological capabilities that are hallmarks of cancer. All these observations on exosomes' role in the delivery of tumorigenic components substantiate their significance in cancer progression and metastasis.

## 5 Functional Roles of Exosomal Lipids in Tumor Microenvironment

### 5.1 Distinctive Lipid Content of Exosomes

**FIGURE 1:**
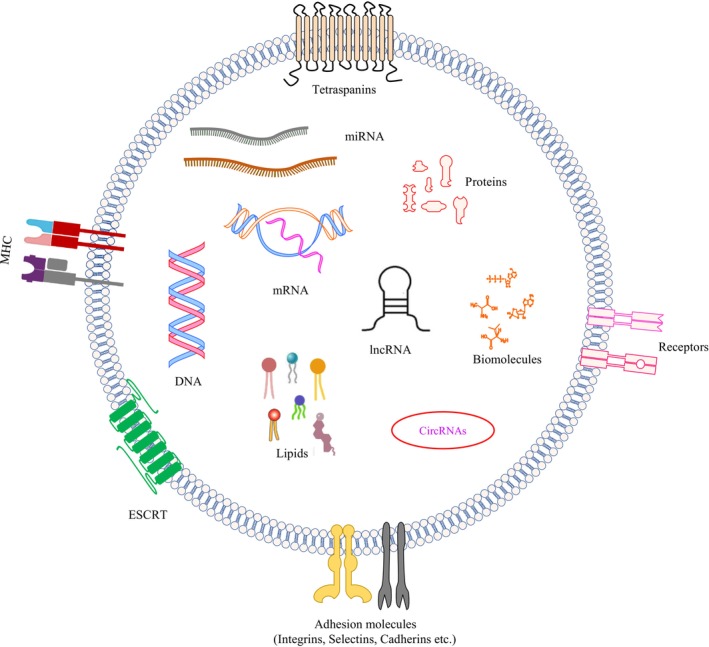
The illustration depicts the lipid cargo of exosomes, enclosed within a heterogeneous lipid bilayer membrane embedded with adhesion molecules and receptors.

The lipids in exosomes differ substantially from those of parental cells. Exosomes' membranes are enriched with SM, gangliosides, and saturated FAs, making them essential structural components of exosomes [99]. The membrane of exosomes is more enriched with PS in their outer leaflet, unlike cellular membranes, where PS is retained in the inner leaflet of the cell plasma membrane, which can function in exosome recognition and internalization [100, 101]. Phosphatidylethanolamine (PE) is randomly but consistently distributed between the two membrane leaflets of exosomes [102]. Exosomes vectorize active enzymes involved in lipid metabolism. For instance, exosomes carry three classes of PLA2: calcium‐dependent PLA2, secreted PLA2, and calcium‐independent PLA2 [103, 104]. Exosomes are enriched with PA compared to parent cells due to the activity of PLD and DGK within the exosomes [64, 105]. Exosomes transport bioactive lipids such as PGs and leukotrienes very efficiently [106, 107]. Thus, exosomes function as lipid carriers and transport bioactive lipids to recipient cells (Figure 1). This trafficking of lipids through exosomes, particularly in the context of the tumor microenvironment, could lead to enhanced tumor progression, metastasis and immunosuppression.

It has been shown that exosomes carry saturated FAs, along with mono‐ and polyunsaturated FAs. A study by Paolino et al. focused on the lipid profiling of CD81 exosomes, a subpopulation of small EVs (sEVs), from melanoma patients and healthy donors (HDs). The research revealed that patients had a higher content of FAs compared to HDs, with notable increases in lauric acid (C12:0), myristic acid (C14:0), palmitic acid (C16:0), stearic acid (C18:0), oleic acid (C18:1), and linoleic acid (C18:2). Among these, oleic acid (C18:1) was the only consistent FA, showing elevated levels in both total and CD81 exosomes from patients compared to HDs. Additionally, total sEVs exhibited a higher prevalence of saturated FAs, primarily palmitic acid (C16:0) and stearic acid (C18:0). In patients, total sEVs showed an increase in unsaturated FAs (C18:1, C18:2n6, C20:4n6) and a decrease in saturated FAs when compared to HDs. This general trend in patient total sEVs reflected a decrease in saturated FAs and an increase in unsaturated FAs. Oleic acid (C18:1), the most abundant unsaturated FA in lipid membranes, was also significantly elevated in CD81 sEVs of stage II and III–IV melanoma [108, 109].

Changes in the lipid composition of exosomes can indicate the presence of cancer. For example, certain lipid species may be more abundant in exosomes derived from cancerous cells compared to non‐cancerous cells. Studies have shown that cancer cells selectively sort specific lipids into exosomes. For example, metastatic PC3 enrich exosomes with glycosphingolipids (GSLs), cholesterol, SM, and PS compared to exosomes of their parental cells. There is limited understanding of how lipids are sorted into exosomes. Research suggests that the sorting mechanisms for proteins and lipids into exosomes operate independently and that lipid sorting could be more closely related to the yield and size of exosomes. U87, Huh7, and MSC are used to test the hypotheses [110]. Proteomic and lipidomic studies showed that protein, not lipid, sorting mechanisms into exosomes depended on the cell type, while lipid sorting into exosomes was related to the size and quantity of exosomes. It was anticipated that the lipid composition of exosomes mirrors that of intraluminal vesicles (ILVs). While the lipid composition of ILVs remains somewhat elusive, they appear to contain higher levels of sphingolipids, cholesterol (CHOL), bis(monoacylglycerol)phosphate (BMP), and phosphatidylinositol (PI)‐3‐phosphate [PI(3)P], compared to the limiting membrane of multivesicular bodies (MVBs) [111, 112].

Additionally, early studies on the role of myelin proteolipid protein (PLP) in lipid sorting revealed that the incorporation of PLP into exosomes is independent of ESCRT mechanisms. Researchers discovered that exosomes containing PLP are enriched with various lipids, including ceramide, which is produced through the activation of SM hydrolysis by nSMase2. Furthermore, inhibiting nSMase2 can reduce the secretion and biogenesis of PLP‐containing exosomes, potentially leading to decreased lipid expression in exosomes [113, 114]. Current research on the mechanisms of lipid sorting is still limited and awaits further exploration.

The specific lipid composition of exosomes can significantly influence their biological functions. Lipids like cholesterol and SM contribute to membrane fluidity and stability, which can affect the exosomes' ability to fuse with target cells and deliver their cargo. This is crucial for processes such as intercellular communication, immune modulation, and tumor microenvironment remodeling. Exosomal lipids, such as glycosphingolipids and PS, act as signaling molecules that can interact with receptors on recipient cells, facilitating various oncogenic processes such as angiogenesis, immune evasion, and metastasis. By analyzing the lipidomic profile of EVs, researchers can monitor the progression of cancer. For instance, a study utilizing matrix‐assisted laser desorption/ionization‐time of flight mass spectrometry data showed a reduction in phosphatidylcholine (PC), PS, PE, phosphatidyl‐inositol, and plasmenylcholine species, alongside an increase in LPC in exosomes from both poorly and highly metastatic melanoma cells. Additionally, exosome lipids revealed a rise in SMs, glycerophospholipids (PA, LPC), and phospholipid bis (monoacylglycero) phosphate, as well as high levels of polyunsaturated PC and plasmenylcholine species, which enhance uptake by neighboring cells [115].

In another case, lipid analysis of exosomes from LIM1215 CRC cells using matrix‐assisted laser desorption/ionization‐time of flight mass spectrometry showed increased levels of sphingolipids, sterol lipids, glycerolipids, and glycerophospholipids, particularly those containing plasmalogens and alkyl ethers [116]. Alterations in exosomes' lipids also occur within lipid subclasses, notably ceramide species, which play a crucial role in cancer. For example, while C18 ceramide species are dominant in the brain, C16 ceramide and C24:1 ceramide are the most prevalent in glioblastoma cells and their derived exosomes, with a significant rise in C24:1 ceramide in mesenchymal cells with semi‐nodular dissemination [117].

The imbalances of exosomal lipids can be observed in biological samples like urine or blood, making them valuable for disease diagnostics. In particular, comparing urinary exosomes from prostate cancer patients with those from PC‐3 cells revealed higher cholesterol and lower phospholipid levels in urinary exosomes, whereas both had similar SM levels. Urinary exosomes had higher PS, while exosomes of PC‐3 cells had higher PC concentrations. Additionally, urinary exosomes exhibited increased hexosylceramide and lactosylceramide species compared to healthy controls, and both urinary exosomes and PC‐3 cells had elevated cholesterol and SM levels [118, 119].

The lipid profiling of exosomes in ovarian cancer showed high concentrations of LPAs such as gangliosides 3, cholesterol ester, acylcarnitines, zymosterol, and specific LPS in exosomes derived from human ovarian cancer cells, SKOV‐3, suggesting their role in ovarian cancer progression [120, 121]. Moreover, exosomes of CC cells showed an enrichment of glycerolipids, sphingolipids, and sterol lipids compared to exosomes of parent cells [116, 122]. Additionally, targeted lipidomic analysis showed different lipid profiles of low and high metastatic triple‐negative breast cancer (TNBC) cell lines. For instance, enrichment of unsaturated DAG 18:1–20:2 was observed in exosomes derived from high metastatic TNBC, while exosomes derived from low metastatic TNBC showed diglyceride 18:0–18:1 [123, 124]. However, the signaling mechanism behind the selective sorting and regulation of the lipid composition of exosomes remains to be elucidated.

Interestingly, FAs of exosomes are utilized as precursors to synthesizing bioactive lipids and stimulating the growth of cancer cells. For instance, arachidonic acid is the precursor of important proliferative and inflammatory modulators, such as eicosanoids and PGs. However, reduced levels of arachidonic acid were found in urine exosomes from prostate cancer patients. It was hypothesized that elevated metabolic products PGE2, 12‐hydroxyeicosatetraenoic acid were detected in malignant prostatic tissue, which resulted in decreased vesicular arachidonic acid levels, as it is utilized in the production of these metabolites [125, 126]. Taken together, these reports suggest the specific sorting and enrichment of certain lipids in exosomes play a role in cancer progression.

### 5.2 Exosomal Lipids Support Cancer Progression and Metastasis

Lipids are essential structural components of exosomes and are involved in oncogenic processes as first and second messengers [127]. A study assessed the role of exosomal lipids in oncogenesis, where synthetic lipid‐enriched nanoparticles that lack proteins and nucleic acids were designed. In pancreatic cancer cells, these nanoparticles stimulated the NF‐κB/SDF‐1α axis, leading to the activation of the AKT‐related cell survival pathway through the binding of the secreted chemokine SDF‐1α to its receptor CXCR4 at the cell surface, which is crucial for cancer progression [41, 128].

Cholesterol is essential for exosomes not only for biogenesis and secretion but also for their membrane stability and uptake. In this context, exosomes from human SOJ‐6 pancreatic tumor cells were found to trigger (glyco)protein ligand‐independent apoptosis and suppress the Notch‐1 pathway in differentiated carcinoma cells, indirectly facilitating the growth of undifferentiated tumor cells. Beloribi et al. proposed that SOJ‐6 exosomes engage with tumor cells through cholesterol‐rich membrane regions, with exosomal lipids playing a crucial role in inducing apoptosis [129].

Managing pain associated with bone cancer remains challenging, as chemotherapeutic drugs often exacerbate the pain. The mechanisms of bone cancer pain involve interactions between cancer cells and nociceptive neurons. Khasabova et al. showed that fibrosarcoma cells express high levels of autotaxin (ATX), an enzyme that synthesizes LPA, which is a pain‐signaling molecule that activates LPA receptors (LPARs) on nociceptive neurons. They explored the role of exosome‐associated ATX‐LPA‐LPAR signaling in the hypersensitivity caused by cancer exosomes. Injecting cancer exosomes intraplantarly into naive mice led to hypersensitivity by sensitizing C‐fiber nociceptors. This study revealed a cancer exosome‐mediated pathway, which could be a potential therapeutic target for managing tumor growth and pain in bone cancer patients [130].

Ferroptosis is a regulated form of cell death characterized by iron‐dependent lipid peroxidation, leading to oxidative damage of cell membranes. Recent studies have shown that inducing ferroptosis in cancer cells can help overcome resistance to traditional therapies like chemotherapy, targeted therapy, and immunotherapy. Exosomes derived from cancer cells can influence ferroptosis by modulating iron metabolism and lipid peroxidation in neighboring cells, contributing to this modulation can contribute to tumor progression or suppression [131]. It has been demonstrated that exosomes derived from adipose tissue decrease ferroptosis susceptibility in CRC, thereby enhancing resistance to oxaliplatin. Researchers discovered that the expression of microsomal triglyceride transfer protein (MTTP) is elevated in the plasma exosomes of CRC patients with a high body fat ratio. This increase in MTTP acts as a ferroptosis inhibitor, diminishing chemotherapy sensitivity. On a molecular level, the MTTP/proline‐rich acidic protein 1 (PRAP1) complex was found to suppress the expression of zinc finger E‐box binding homeobox 1, while boosting the levels of glutathione peroxidase 4 and xCT. This MTTp/PRAP1/ZEB1 axis‐mediated inhibition of ferroptosis led to a reduction in the polyunsaturated FAs ratio and lipid ROS levels [132]. Lipid peroxidation is a key feature of ferroptosis. Yang et al. investigated how glioblastoma‐derived exosomes (GDEs) affect lipid accumulation and oxidation in dendritic cells (DCs). The FAs present in GDEs prompt a metabolic shift toward oxidative phosphorylation, leading to immune dysfunction in DCs. When bone marrow‐derived dendritic cells (BMDCs) were treated with GDEs, their viability decreased compared to immature dendritic cells (imDCs). This treatment triggered ferroptosis in mature dendritic cells (mDCs) via the NRF2/GPX4 pathway, thereby promoting glioblastoma growth [133].

In addition, it was observed that unsaturated DAGs are enriched in exosomes from highly TNBC. DAG‐mediated protein kinase C activation occurs in many oncogenic processes. The accumulation of DAGs in exosomes can activate the protein kinase D signaling cascade in endothelial cells, facilitate angiogenesis, tumor metastasis, and progression. It was also proved that PLD/PLCμ phosphorylation was induced by the biological activity of exosomes in endothelial cells, leading to neo‐angiogenesis [134]. This demonstrates the selective sorting of DAG lipid species into exosomes and their role in cancer progression and metastasis. Conversely, exosomes derived from prostate cancer patients showed a significant increase in PG 22:6/22:6 and a decrease in neutral lipids, suggesting a shift toward increased energy consumption by PC3 mediated through exosomes [109, 135].

### 5.3 Exosomal Lipids in Immunomodulation During Cancer Progression and Metastasis

Immunomodulation is pivotal to tumor metastasis. Interestingly, exosomes mediated immunomodulation in tumors has been widely reported. For instance, RNAs of exosomes trigger lung alveolar epithelial cells via Toll‐like receptor 3 and promote the recruitment of neutrophils and the formation of a lung metastatic niche [136]. It was shown that tumor‐derived exosomes of pancreatic cancer cells deliver TGF‐β1 to natural killer (NK) cells, contributing to immunomodulation mediated through Smad2/3 phosphorylation and downregulation of NKG2D in NK cells [137]. Lipids of exosomes, which have roles in structural and metabolic remodeling, likely have immunomodulatory functions as well [138]. For example, sphingosine can mediate the structural properties of exosomes, and sphingosine is converted into S1P enzymatically in exosomes of ovarian cancer, which is involved in the reduction of cytotoxicity of T cells [139]. It was reported that exosomes derived from solid tumors of ovarian cancer cells expressed PS, which engaged in abrupt and reversible blocking of T cell receptor activity in CD4^+^ and CD8^+^ T cells mediated through the DAG kinase inhibition of DAG. These findings suggest the therapeutic significance of exosomes that express PS by applying DAG kinase inhibitors to increase T‐cell responses in tumors [140].

Notably, glycans have a crucial role in cancer immunomodulation, but the glycolipids of exosomes remain largely unexplored [141]. Besides T cell suppression, the lipids of exosomes derived from tumor cells are involved in dendritic cell destruction. Of note, a metabolic shift toward oxidative phosphorylation with excess LD biosynthesis by exosome‐associated free FAs resulted in dendritic cell immune suppression [142]. Interestingly, a few reports showed that the enrichment of PGE2 has a predominant role in promoting tumor growth by triggering coordinated immunosuppression [143, 144]. Overall, these findings suggest that the lipids of exosomes play a role in cancer immune evasion. However, further investigation is indispensable to uncover the impact of exosomal lipids on the signaling of cancer progression.

### 5.4 Exosomal Lipids as an Energy *Source:* Another Driver of Metastasis

Several groups have studied the role of FAs in exosomes in cancer. The CD81sEVs (CD81‐expressing small EVs) showed that many FAs were enriched in exosomes derived from cancer patients compared to those obtained from healthy donors. These CD81sEVs with high levels of lauric (C12:0) and myristic acids (C14:0) may be related to the accelerated metabolism of lipids in advanced cancer [108, 145]. Triglycerides (TGs) are known as energy storage molecules [146]. It was reported that, as compared to exosomes of tuberculosis pleural effusion, TGs have been found more in exosomes from malignant pleural effusion, suggesting their role in malignancy [147].

Recently, β‐oxidation of FAs has been gaining attention as an energy source during tumor development. Interestingly, it was observed that FAs in exosomes can provide energy through β‐oxidation and accelerate lung tumorigenesis [148]. The hypoxic exosomes from PC3 transfer palmitic and oleic FAs, which generate ATP in mitochondria. These FAs can be utilized as fuel in the hyperoxic recipient cells at the edge of tumors, thus influencing the aggressiveness of the tumor [149]. Additionally, AT‐exosomes derived from adipocytes can provide exogenous FAs and FAO‐associated proteins to stimulate FAO in melanoma [150]. Similarly, adipocyte‐derived lipids and proteins are involved in FAO in various cancers, suggesting that adipocyte exosomes facilitate ATP production in tumor cells [93, 151]. These studies highlight the significance of FAs and FAO in exosomes for ATP production and the survival of cancer cells.

## 6 Conclusion

Tumor growth requires lipid metabolic reprogramming, which includes increased lipid uptake/storage/synthesis, as well as FAO. These processes are regarded as significant hallmarks of cancer. In addition, studies have revealed that cancer cell‐derived exosomes modulate various oncogenic processes. Despite being the major cargo of exosomes, the role of lipids in cancer progression remains poorly understood. Recent studies have demonstrated that exosomal lipids serve as signaling molecules, energy sources, and membrane structures, facilitating cancer metastasis and progression. However, understanding the mechanisms of lipid transfer from exosomes to cancer cells is crucial for targeted therapies. The specific roles of different lipid species in different cell signaling and their roles in cancer progression need further elucidation. Clinical translation requires developing reliable biomarkers and strategies to target exosomal lipids effectively. The tumor microenvironment, including adipocyte interactions, influences exosome lipid composition, necessitating further research. Addressing these challenges will enhance our understanding of the role of lipids in tumor‐derived exosomes, holds great promise for novel therapeutic interventions that complement current approaches and improve cancer treatment outcomes.

## Author Contributions

**Dandugudumula Ramu:** conceptualization, writing – original draft, data curation, writing – review and editing. **Eunjoo Kim:** supervision.

## Conflicts of Interest

The authors declare no conflicts of interest.

## Data Availability

There are no data associated with this research.
